# Role of the Cytokinin-Activated Type-B Response Regulators in Hormone Crosstalk

**DOI:** 10.3390/plants9020166

**Published:** 2020-01-30

**Authors:** Yan O. Zubo, G. Eric Schaller

**Affiliations:** Dartmouth College, Department of Biological Sciences, Hanover, NH 03755, USA; yan.o.zubo@dartmouth.edu

**Keywords:** cytokinin, type-B ARR, ChIP-seq, phytohormone, two-component system, multistep phosphorelay, crosstalk, transcription

## Abstract

Cytokinin is an important phytohormone that employs a multistep phosphorelay to transduce the signal from receptors to the nucleus, culminating in activation of type-B response regulators which function as transcription factors. Recent chromatin immunoprecipitation-sequencing (ChIP-seq) studies have identified targets of type-B ARABIDOPSIS RESPONSE REGULATORs (ARRs) and integrated these into the cytokinin-activated transcriptional network. Primary targets of the type-B ARRs are enriched for genes involved in hormonal regulation, emphasizing the extensive crosstalk that can occur between cytokinin, auxin, abscisic acid, brassinosteroids, gibberellic acid, ethylene, jasmonic acid, and salicylic acid. Examination of hormone-related targets reveals multiple regulatory points including biosynthesis, degradation/inactivation, transport, and signal transduction. Here, we consider this early response to cytokinin in terms of the hormones involved, points of regulatory crosstalk, and physiological significance.

## 1. Introduction

Cytokinins are a class of phytohormones that regulate multiple aspects of plant growth and development including shoot and root growth, chloroplast development, seed fill, senescence, and nutrient uptake [1,2,3,4,5]. They also regulate responses to the environment such as to abiotic and biotic stresses [2,3,6,7,8]. Cytokinin signal transduction occurs through a multistep phosphorelay related to the two-component signaling systems of prokaryotes, and incorporates receptors with histidine–kinase activity, phosphotransfer proteins, and type-B response regulators, these last functioning as transcription factors to regulate gene expression [2,3]. Cytokinin signal transduction has been most thoroughly characterized in Arabidopsis which employs three ARABIDOPSIS HISTIDINE KINASES (AHKs) as cytokinin receptors, these being predominantly localized to the endoplasmic reticulum membrane; five ARABIDOPSIS HISTIDINE-CONTAINING PHOSPHOTRANSFER PROTEINs (AHPs) which cycle between the cytosol and the nucleus; and eleven type-B ARABIDOPSIS RESPONSE REGULATORs (ARRs) which mediate the primary transcriptional response to cytokinin [2,3,9]. Among the well-characterized cytokinin primary-response genes are the type-A *ARR*s which encode a second family of response regulators that lack a DNA-binding motif and negatively regulate cytokinin responses [10,11,12]. Similar families of cytokinin signaling elements have been identified and functionally characterized in other plant species with phylogenetic analysis indicating that a complete cytokinin signaling pathway first appeared in basal land plants such as the moss *Physcomitrella patens* [13,14].

The type-B response regulators have a modular structure. They have a receiver domain, the diagnostic domain of a response regulator, at their N-terminus. The receiver domain contains a conserved Asp residue which serves as the site for regulatory phosphorylation when the multistep phosphorelay is activated [15,16,17,18,19,20]. Type-B response regulators also have C-terminal extensions of variable length, all of which contain a conserved Myb-like DNA-binding domain [18,21]. The Myb-like DNA-binding domain is often referred to as a GARP domain, so named based on sequence conservation between the maize GOLDEN2, Arabidopsis type-B ARR, *Chlamydomonas reinhardtii* PSR1, and Arabidopsis PHR1 transcription factors [18,21]. The Myb-like domain of the type-B ARRs binds to a short (A/G) GAT core DNA sequence that is critical and sufficient for type-B ARR binding [21] but optimal binding is to extended DNA sequences containing this core based on analysis using protein-binding microarrays [22,23,24]. For example, the type-B response regulator ARR1 of Arabidopsis binds with high affinity to primary (AGATACGG), secondary (AAGATCTT), and tertiary (CGGATCCG) DNA motifs, all of which contain the 4 base core sequences [24]. The modular structure of the type-B response regulators is likely to play an important role in regulating their activity based on the current model [21,24,25,26,27]. According to this model, the receiver domain inhibits DNA binding by the response regulator in its non-phosphorylated state. Phosphorylation of the conserved Asp in the receiver domain, such as in the response to cytokinin, relieves the inhibition and exposes the Myb-like DNA-binding motif, allowing the type-B response regulator to bind to its targets and initiate transcription.

Although Arabidopsis contains 11 type-B ARRs, these do not all contribute equally in cytokinin signal transduction [25,28,29,30]. Genetic analysis, based on loss-of-function mutations in the various type-B ARR family members, indicates that ARR1, ARR10, and ARR12 play predominant roles in regulating transcriptional and physiological responses to cytokinin [28,29,30,31]. In particular, the triple mutant *arr1 arr10 arr12* abolishes the majority of cytokinin-mediated gene expression as well as rendering the plant largely insensitive to cytokinin based on a variety of physiological assays [29,30]. Through transcriptome analysis, a core set of early response genes have been identified in Arabidopsis, referred to as the Golden List, whose expression is consistently up- or downregulated in response to cytokinin [32,33]. Not surprising, the type-A *ARR*s, which are primary response genes that negatively regulate cytokinin responses, are among the most uniformly upregulated members of the Golden List [10,11,12,33]. The type-B response regulators thus regulate the expression of cytokinin primary-response genes that would serve as their direct targets and also of additional non-target genes whose expression is dependent on that initial cytokinin transcriptional response. Furthermore, the type-B response regulators, in addition to initiating the transcriptional response to cytokinin, also initiate a negative feedback loop that desensitizes the plant to cytokinin.

Recently, two complementary Chromatin Immunoprecipitation-sequencing (ChIP-seq) studies were carried out with type-B ARRs of Arabidopsis, these studies providing the first genome-wide views of the type-B ARR targets involved in the initiation of the cytokinin response [24,34]. Results from these analyses, coupled to transcriptome data, shed light on the means by which cytokinin regulates growth and development as well as responses to biotic and abiotic factors. Of particular interest is the finding that primary targets of the type-B ARRs are enriched for genes involved in hormonal regulation, emphasizing the extensive crosstalk that can occur between cytokinin, auxin, abscisic acid, brassinosteroids, gibberellic acid, ethylene, jasmonic acid, and salicylic acid [8,35,36,37,38,39]. Examination of hormone-related targets of the type-B ARRs reveals multiple regulatory points, including biosynthesis, degradation/inactivation, transport, and signal transduction. Here, we consider this primary response to cytokinin in terms of the hormones involved, points of regulatory crosstalk, and physiological significance.

## 2. Characteristics of the type-B ARR ChIP-seq Studies

Two complementary ChIP-seq studies were carried out by Zubo et al. [24] and Xie et al. [34] to identify targets of the Arabidopsis type-B ARRs that initiate the primary response to cytokinin [24,34]. Zubo et al. [24] ectopically overexpressed GFP-tagged ARR10 in the *arr1 arr10 arr12* cytokinin-insensitive mutant line, the *35S:ARR10-GFP* construct complementing a variety of *arr1 arr10 arr12* mutant phenotypes and resulting in a hypersensitive cytokinin response [24]. These data were consistent with *35S:ARR10-GFP* rescuing the loss of not only the native *ARR10* activity but that of all three type-B ARRs (*ARR1*, *10*, and *12*). The cytokinin-hypersensitivity, coupled to the lack of competition from ARR1 and ARR12, were predicted to increase the sensitivity of these lines for identifying targets of ARR10 by ChIP-seq. Cytokinin treatment was performed on ~3 week old green seedlings for 30 min with 5 µM of the cytokinin benzyladenine (BA) [24], BA having previously been determined to induce gene expression within 10 min and some known primary response genes reaching maximal induction within 2 h [40,41,42]. Three ChIP-Seq biological replicates for the cytokinin treatment were made (two from one transgenic line and one from a second independent transgenic line), and ARR10 binding peaks were defined based on conservation of their summit position in all three replicates. Using the conserved binding peaks, 4004 “candidate gene targets” were identified, and within this group, 804 were “gene targets” based on transcriptome analyses for cytokinin-regulated genes [24].

The ChIP-seq approach taken by Xie et al. [34] differed from the approach taken by Zubo et al. [24] in three key respects: method for transgene expression; age of seedlings used; and duration of cytokinin treatment [24,34]. Xie et al. [34] focused on a recombineering approach to drive expression of YFP-tagged versions of *ARR1*, *ARR10*, and *ARR12* and which mimics, as closely as possible, the native expression state of these genes both in terms of expression levels and organ-specificity [34]. The recombineered type-B ARR transgenes were expressed in the *arr1 arr10 arr12* mutant line, the same line exploited by Zubo et al. [24], each transgene rescuing the aborted root growth phenotype of the mutant demonstrating their functionality [34]. The recombineered lines are predicted to have reduced cytokinin sensitivity compared to the wild type, as each would still be double mutant for the loss-of-function mutations not complemented by the transgene, the double mutants involving the *arr1*, *arr10*, and *arr12* alleles all being cytokinin hyposensitive to varying extents [28,29,30]. However, as with the study by Zubo et al. [24], the reduced competition from the other type-B ARRs is likely to increase the sensitivity of the recombineered lines for identifying targets of the tagged type-B ARRs by ChIP-seq. Xie et al. [34] performed ChIP-seq using 3 day old green seedlings as compared to the 2–3 week old green seedlings used by Zubo et al. [24]; thus, the seedlings examined by Xie et al. [34] would have a substantially higher proportion of embryonic tissues (cotyledons and primary root) than those examined by Zubo et al. (2017) [24,34]. Xie et al. [34] used a similar cytokinin concentration (10 µM BA) to that employed by Zubo et al. [24] (5 µM BA), but the cytokinin treatment was for 4 h as compared to 30 min [24,34]. Xie et al. [34] performed single cytokinin treatment for ChIP-seq of the recombineered *ARR1*, *ARR10*, and *ARR12* lines, allowing them to identify candidate gene targets for each type-B ARR [34]. From these datasets, Xie et al. [34] compiled a dataset of 8770 candidate genes representing the “union” of the individual datasets and, to achieve the higher fidelity of biological replication, also compiled a dataset of 3373 “common” candidate genes based on these genes being identified in the ChIP-seq datasets for ARR1, ARR10, and ARR12 [34]. Xie et al. [34] identified 813 targets from their “common” dataset and 1713 targets from their “union” dataset based on these being differentially expressed in cytokinin transcriptomic analyses [34].

In spite of the differences in experimental design—transgenic expression strategy, age of seedlings, duration of cytokinin treatment—there is a great deal of consistency in the ChIP-seq results obtained in the two studies [24,34]. First, in both studies, the ChIP-seq binding sites are enriched for DNA-binding motifs containing a core (A/G) GAT sequence independently determined as critical and sufficient for type-B ARR binding [21,22,23,24,34]. Second, the type-B ARR binding sites are enriched near transcription start sites, consistent with predictions for the classical regulation of gene expression. Third, gene ontology (GO) analysis of the candidate target and target genes revealed similar sets of enriched genes including the general categories for responses to light, abiotic stimuli, organic substances, and hormone stimuli as well as more specific subcategories within these such as responses to the various hormone stimuli indicative of crosstalk that we focus on below.

**Figure 1:**
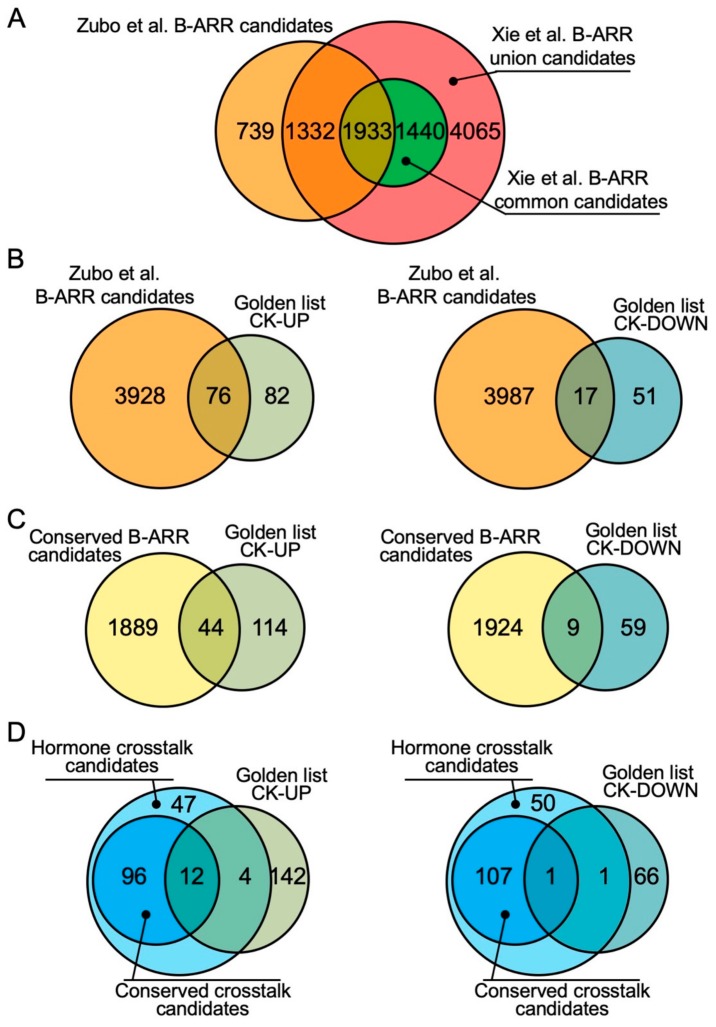
Comparative analysis of the type-B ARABIDOPSIS RESPONSE REGULATOR (ARR) Chromatin Immunoprecipitation-sequencing (ChIP-seq) datasets. (**A**) Venn diagram comparing B-ARR candidate genes from Zubo et al. [24] to Xie et al. [34]. (**B**) Venn diagrams comparing Zubo et al. [24] B-ARR candidate genes to the Golden List of cytokinin (CK)-regulated genes [33]. (**C**) Venn diagrams comparing the conserved B-ARR candidates to the Golden List of CK-regulated genes. (**D**) Venn diagrams comparing hormone crosstalk candidates from Zubo et al. [24], “conserved” crosstalk candidates, and the Golden List of CK-regulated genes.

Comparison of the dataset of the 4004 candidate genes identified by Zubo et al. [24] to the datasets compiled by Xie et al. [34] is consistent with the ectopically expressed ARR10 largely fulfilling the role of ARR1, ARR10, and ARR12 in regulating the primary transcriptional response to cytokinin. For example, 69.5% of the Zubo et al. [24] type-B ARR candidates (2783 out of 4004) are found in the Xie et al. [34] ARR10 candidates with this percentile reaching 81.5% (3265 out of 4004) when compared to the Xie et al. [34] “union” candidates (3265 out of 4004) (Figure 1A). From the datasets identified by the two studies, we can also define a highly conserved subset of 1933 candidate target genes found in all six cytokinin-treated biological replicates (“conserved B-ARR candidates”), this set representing the intersection of the Zubo et al. [24] candidates (three biological replicates) and the Xie et al. [34] “common” candidates (three biological replicates) (Figure 1A; Supplementary Materials Table S1).

Comparison of the type-B ARR candidate targets to the robustly cytokinin-regulated genes—the Golden List [33]—is also revealing. The Golden List consists of 158 genes that are consistently upregulated and 68 genes that are consistently downregulated by cytokinin based on transcriptomic studies [33]. The Zubo et al. [24] B-ARR candidates correspond to 48% of the upregulated and 25% of downregulated genes from the Golden List (Figure 1B). Similarly, for the conserved B-ARR candidates, there is a greater correspondence to the upregulated genes (28%) than to the downregulated genes (14%) from the Golden List (Figure 1C). We defined this set of differentially expressed genes as the “Golden type-B ARR targets” (Supplementary Materials Table S2) based on their being consistent primary targets for the type-B ARRs and resulting in rapid up- or downregulation for their expression in response to cytokinin. The promoters of the upregulated genes from the Golden List are enriched with extended type-B ARR DNA-binding motifs (i.e., sequences longer than the core 4 bp DNA sequence sufficient for type-B ARR binding) as elucidated by the use of protein-binding microarrays which is likely to confer higher affinity of the type-B ARRs for these targets and, therefore, result in their rapid and reproducible transcriptional response to cytokinin [21,22,23,24].

## 3. Hormone Crosstalk Revealed by ChIP-seq Studies

**Figure 2:**
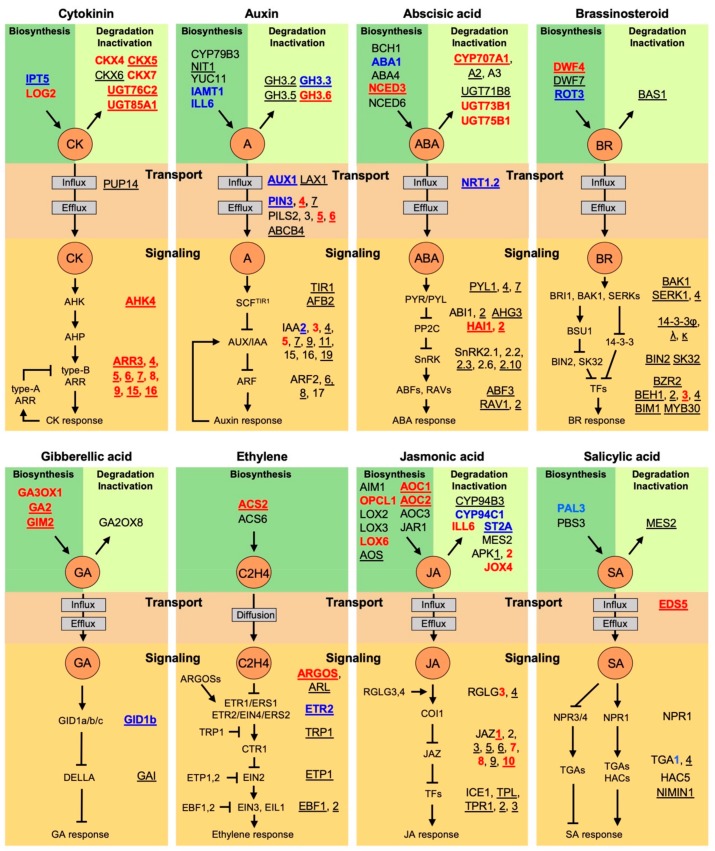
Cytokinin regulates hormone crosstalk through action of the type-B ARRs, pathways by which activity of the phytohormones cytokinin, auxin, abscisic acid, brassinosteroid, gibberellic acid, ethylene, jasmonic acid, and salicylic acid are outlined. Type-B ARR candidate genes involved in hormone biosynthesis, degradation/inactivation, signaling, and transport are indicated based on the Zubo et al. [24] dataset. Genes are underlined if part of the “conserved crosstalk” dataset based on the intersection of the Zubo et al. [24] candidate genes with the Xie et al. [34] “common” candidate genes. Type-B ARR targets confirmed by expression are in bold and highlighted in red if induced by cytokinin or blue if inhibited by cytokinin.

Gene Ontology (GO) analysis reveals that the primary targets of the type-B ARRs are enriched genes involved in various hormone responses, emphasizing the extensive hormone crosstalk mediated through action of cytokinin and the type-B ARRs [24,34]. Examination of these hormone-related targets reveals multiple regulatory points, including biosynthesis, degradation/inactivation, transport, and signal transduction, indicating the concerted action of cytokinin in regulating such crosstalk. In the subsections below, we consider these targets in terms of feedback regulation of cytokinin on its own activity, as well as points of crosstalk with auxin, abscisic acid, brassinosteroids, gibberellic acid, ethylene, jasmonic acid, and salicylic acid. In considering these major points of crosstalk, we make use of the candidate type-B ARR targets identified by Zubo et al. [24], these identifying over 150 genes involved in the regulation of phytohormone activity and referred to below as “hormone crosstalk candidates” (Figure 1D, Figure 2, Supplementary Materials Table S3). Additionally, approximately two-thirds of these genes are contained within the more restrictive set of conserved B-ARR candidates (Figure 1D, Figure 2, Supplementary Materials Table S3), these defined as the intersection of the Zubo et al. [24] candidates with the Xie et al. [34] “common” candidates and referred to below as “conserved crosstalk candidates”; these conserved crosstalk candidates are underlined in Figure 2.

Of particular interest are those genes for which expression information is available for their response to cytokinin. These type-B ARR target genes, based on transcriptomic studies to identify cytokinin-responsive genes [24,30,33], are indicated by highlighting in Figure 2, red indicating their upregulation and blue their downregulation by cytokinin. Cytokinin transcriptomic studies, not surprisingly, focus primarily on the early transcriptional response to cytokinin [24,33]. Thus, although there are type-B ARR candidate targets for which no changes in expression are indicated, many are still likely to be of physiological significance. These may represent genes that exhibit slower kinetics for their transcriptional response to cytokinin, exhibit a response only under specific conditions or in certain tissues, and/or require combinatorial interactions with other transcription factors for their regulation. Consistent with Type-B ARR candidate targets being of physiological significance is that they exhibit similar GO enrichment to that found for targets with verified expression information [24]. Similarly, the key shoot meristem regulator *WUSCHEL* is a type-B ARR target transcriptionally induced by cytokinin, although it does not generally appear in the transcriptomic studies, this likely being because *WUSCHEL* expression occurs predominantly in a subset of meristem cells and also exhibits a long-term kinetic response to cytokinin [24,34,43,44].

### 3.1. Cytokinin Negative Feedback Regulation

Prior studies indicate that one transcriptional response to cytokinin is the induction of genes that negatively regulate cytokinin activity [10,11,33,40,45,46,47]. These genes include those that encode enzymes to degrade or inactivate cytokinins: the *CKX*s which encode cytokinin oxidases and the *UGT*s which encode glucosyl transferases for conjugation of glucose to cytokinin [5,48,49]. The gene *CKX4* was identified as a primary response gene regulated by the type-B ARR and ARR1 [47]. These negative regulatory genes also include the type-A *ARR*s [10,11,40,45,46] which were previously identified as cytokinin primary response genes based on their induction by cytokinin in the presence of cycloheximide [10,47]. The type-A ARRs are response regulators containing a phosphorylatable receiver domain, and so one mechanism by which they may downregulate signaling by the pathway is to compete with the type-B ARRs for phosphorylation by AHPs; this will channel the multistep phosphorelay away from the type-B ARR transcriptional regulators to reduce their effects on gene expression [3,50,51]. However, given the family size of the type-A ARRs (10 family members in Arabidopsis), one might also hypothesize that other mechanisms come into play, this possibility being supported by the finding that a type-A ARR phosphomimic still exhibits functionality, even though it cannot be phosphorylated and so should not compete with the type-B ARRs [12]. Additionally, the induction of type-A ARRs has been implicated in mediating the antagonistic interaction of cytokinin with the abscisic acid (ABA) signaling pathway [52,53].

Based on expression analysis, cytokinin induces multiple negative feedback loops to regulate both cytokinin metabolism and signal transduction with such negative feedback loops being a common feature of hormone responses to allow for inactivation as well as adaptation so that the organism can respond to increasing hormone concentrations [54,55]. The ChIP-seq studies provide direct evidence that many of these genes are primary targets for the type-B ARRs (Figure 2) [24,34]. In terms of cytokinin metabolism, these genes include multiple members of the *CKX* and *UGT* families for the degradation and inactivation of cytokinins as well as the biosynthetic gene *IPT5*, the expression of which is downregulated in response to cytokinin [56]. In terms of signal transduction, these primary targets include the family of type-A *ARR*s, members of the type-A *ARR* family exhibiting some of the strongest binding by the type-B ARRs along with a pronounced cytokinin dependence for this binding [24,34]. Not only does ChIP-seq identify these genes as primary targets, the majority of these also have transcriptomic expression information associated with them (Figure 2), consistent with the initial binding of the type-B ARRs serving to rapidly regulate changes in their expression to negatively regulate cytokinin activity.

Although the majority of type-B ARR primary targets are consistent with negative regulation of cytokinin activity, there are two potential exceptions. First, the cytokinin biosynthetic gene *LOG2* is a target that is upregulated in response to cytokinin, potentially serving to help replenish the cytokinin pool, although this is not a family member implicated in playing a major role in either the shoot apical meristem or the root [57]. Second, *AHK4*, which encodes a cytokinin receptor is also a primary target upregulated in response to cytokinin. However, AHK4, like many histidine kinases, possesses both histidine kinase and phosphatase activity and so has the potential to function as both a positive and a negative regulator of the pathway, dependent on cytokinin levels [58].

### 3.2. Cytokinin-Auxin Crosstalk

Auxin controls fundamental aspects of plant growth, organogenesis, and responses to the environment [59,60,61,62]. Based on its critical role in plant growth and development, auxin is one of the most intensively studied of the phytohormones, including its interaction and crosstalk with other phytohormones such as cytokinin [60,63,64]. Although the regulatory interactions between auxin and cytokinin are often considered antagonistic, the truth is more complicated and involves both antagonistic and supportive roles for crosstalk between these hormones, often in a cell and/or tissue-specific manner, and with the timing of such interactions also being of critical importance. Gene expression studies reveal multiple points of crosstalk between auxin and cytokinin with both hormones capable of regulating each other’s activity through effects on metabolism, transport, and signaling [33,60,63]. A thorough discussion of crosstalk between these hormones is a beyond the scope of this review, but a few examples point to the complexity possible and its repercussions. Relating to auxin’s ability to regulate cytokinin activity, auxin induces the transcription of the type-A *ARR*s *ARR7* and *ARR15* to inhibit the cytokinin response during Arabidopsis root embryogenesis [65]. However, in another tissue—the shoot meristem—auxin represses expression of type-A ARRs to increase the sensitivity of these cells to cytokinin [66]. Relating to cytokinin’s ability to regulate auxin activity, cytokinin induces expression of the *AUX/IAA* gene *SHY2/IAA3* which functions as a negative regulator of auxin signaling to inhibit auxin responses and cell proliferation in the primary root [60,64,67,68]. Cytokinin also inhibits expression of the *AUX1* auxin efflux carrier, affecting the shootward movement of auxin from the root tip and resulting in decreased root cell expansion [69].

The ChIP-seq studies indicate that the type-B ARRs target genes are involved in auxin metabolism, transport, and signal transduction (Figure 2). Expression analysis confirms cytokinin-dependent changes in expression from a number of genes in each category, but the list of candidate targets greatly extends the number of potential regulatory targets by which cytokinin may crosstalk with auxin. Auxin metabolism targets include genes involved in both auxin biosynthesis and inactivation. Cytokinin suppresses the expression of the biosynthetic genes *IAMT1* and *ILL6* and induces the expression of *GH3.6* which encodes an acyl acid amido synthetase for the inactivation of auxin by conjugation to amino acids, consistent with their being primary targets and indicating that cytokinin would likely reduce the levels of available auxin. Interestingly, although the four *GH3* genes involved in auxin inactivation are all conserved type-B ARR candidates, of the five auxin biosynthesis genes, only *NIT1* is a conserved B-ARR candidate. However, in the study by Xie et al. [34], *CYP79B3* was bound by ARR1 after 3 h cytokinin treatment, and *ILL6*, *IAMT1*, and *CYP79B3* all bound by ARR1 after three days of treatment; additionally, *IAMT1* was bound by ARR10 without cytokinin treatment [34]. This may suggest some native specificity among the type-B ARRs in the regulation of these biosynthetic genes. Although the type-B ARRs exhibit overlapping expression profiles and can substitute for each other based on many physiological responses, they exhibit differences in their native expression levels and tissue prevalence which can lend to specificity members of the type-B ARR family [34,70,71,72].

Genes related to auxin transport, including both efflux and influx carriers, are represented among the B-ARR targets and candidate targets (Figure 2). The PIN auxin efflux carriers have a major role in the polar transport of auxin and establishing distribution of auxin in various cells and tissues to control growth and development [73,74,75]. The PILS auxin efflux carriers are also well represented and may be of particular interest, their role in the regulation of auxin activity not nearly as well understood as that of the PINs. Unlike the PIN carriers, most of which are found at the plasma membrane and, therefore, regulate the cell to cell movement of auxin and its distribution with tissues, the PILS carriers are localized to the endoplasmic reticulum (ER) with a potential role of sequestering auxin within the ER lumen [73,74,75]. The AUX1/LAX gene family encodes auxin influx carriers. Expression of *AUX1* is downregulated in response to cytokinin, the cytokinin-dependent peak(s) for type-B ARR binding being found in the eighth intron of AUX1 [24,69]. We note that AUX1 does not appear in the list of candidate targets from Zubo et al. [24], this being due to the restriction of having peak summits within close proximity in the three biological replicates; however, robust binding peaks are present in all three replicates based on visual inspection [24].

Type-B ARR binding sites are also found associated with genes encoding elements of the auxin signal transduction pathway, including two genes encoding auxin receptors, ten *AUX/IAA* genes, and four *ARF* genes. Cytokinin-dependent expression information is only available for a few of these, the most significant being for *SHY2/IAA*3, a negative regulator of auxin activity. The gene *SHY2/IAA*3 is induced in response to cytokinin and plays a pivotal role in regulating the balance of auxin to cytokinin activity in the root, its induction by cytokinin reducing auxin activity and resulting in a decrease in cell proliferation by the primary root [60,64,67,68].

### 3.3. Cytokinin-Abscisic Acid Crosstalk

Abscisic acid (ABA) regulates plant responses to such abiotic stresses as drought and cold [76,77,78]. Abscisic acid also regulates various developmental processes including dormancy, stomatal closure, and fruit development [76,79,80,81]. Crosstalk between cytokinin and ABA is frequently antagonistic. Increased cytokinin levels suppress ABA responses, and multiple ABA-responsive genes are upregulated in the cytokinin receptor *ahk2 ahk3* double mutant [53,82]. Antagonistic physiological interactions have been found in the regulation of the Arabidopsis drought stress response, based in part on genetic analysis of *AHK* cytokinin receptors and the type-A and type-B ARRs involved in cytokinin signaling and the SnRK2 kinases involved in ABA signaling [82,83,84]. The type-A *ARR*s, which are rapidly induced cytokinin primary response genes, have been identified as key players mediating this antagonistic interaction in several instances. For instance, the ability of ABA to inhibit seed germination and cotyledon greening requires the ABA-activated transcription factor ABI4 to suppress transcription of type-A *ARR*s [52]. Additionally, type-A ARRs physically interact with the transcription factor *ABI5*, as well as negatively regulate its expression, *ABI5* being rapidly upregulated in response to ABA and playing a key role in mediating the ABA response [53].

Type-B ARR binding sites are found associated with genes involved in biosynthesis, degradation/inactivation, transport, and signaling (Figure 2). Based on cytokinin-dependent changes in expression, the majority would result in decreased ABA activity. The type-B ARR targets predicted to downregulate ABA activity in response to cytokinin include: (1) the inhibition of the ABA biosynthesis gene *ABA1*; (2) the induction of ABA degradation/inactivation genes such as *CYP707A1,* which encodes an hydroxylase involved in ABA catabolism, and the *UGT*s, which encode UDP glucosyl transferases, involved in conjugation of glucose to ABA; (3) the downregulation of *NRT1.2* for ABA influx; and (4) the upregulation of HAI1 and HAI2, which encode PP2C phosphatases that negatively regulate ABA signaling. We note that although many of the candidate targets involved in metabolism are not among the “conserved” B-ARR dataset, the study by Xie et al. [34] does support ARR1 binding to *BCH1* and *ABA1*, and ARR12 binding to *ABA4* and *NCED6*.

### 3.4. Cytokinin-Brassinosteroid Crosstalk

Brassinosteroids are polyhydroxylated steroid hormones that regulate various aspects of plant growth and development, such as cell proliferation and expansion, vascular differentiation, reproduction, photomorphogenesis, and senescence [85,86,87]. Cytokinin and brassinosteroids can regulate processes antagonistically or cooperatively, acting antagonistically in the regulation of leaf senescence and cooperatively in the stimulation of cell proliferation as well as the biosynthesis of ethylene and anthocyanin [88,89,90,91]. Interestingly, both cytokinin and brassinosteroids stimulate cell proliferation by up-regulating transcription of the *CycD3* gene, and in some cases brassinosteroids can substitute for cytokinin to promote cell proliferation in Arabidopsis callus and suspension cells [92]. In another study, treatment of wheat seedlings with 24-epibrassinolide inhibited the expression and activity of cytokinin oxidases (CKXs), key cytokinin degradation enzymes, and elevated the cytokinin level [93].

Type-B ARR binding sites are found associated with genes involved in brassinosteroid biosynthesis and degradation and multiple genes involved in brassinosteroid signaling, including brassinosteroid perception (*BAK1*, *SERK1,4*), signal transduction (*BIN2*, *SK32*, and *14-3-3* proteins), and transcriptional regulation (*BZR2*, *BEH1, 2, 3, 4*, *BIM1*, *MYB30*) (Figure 2). However, only a few of the genes involved in brassinosteroid signaling exhibited transcriptional changes in response to cytokinin, potentially due to the temporal/spatial mode of these genes’ regulation. Nevertheless, brassinosteroid-related genes exhibited some of the highest level of reliability among hormone-related genes for type-B ARR binding: all 19 of the genes are members of the “conserved” B-ARR candidates.

### 3.5. Cytokinin-Gibberellic Acid Crosstalk

Gibberellins are plant hormones that play key roles in developmental programs including seed germination, stem elongation, flowering, trichome development, and leaf expansion [94,95,96]. Generally, gibberellic acid and cytokinin are viewed as antagonists, as they have an opposing effects on the shoot apical-meristem formation, root growth, and hypocotyl elongation [97,98]. One mechanism by which such antagonistic hormone crosstalk can occur was recently uncovered in the analysis of the DELLA proteins which function as negative regulators of gibberellin signaling (Figure 2); the DELLA proteins GAI and RGA directly interact with the type-B ARR ARR1 to act as transcription co-activators to control root development and photomorphogenic traits [99]. On the other hand, both hormones can promote male organ development in Arabidopsis and tobacco [100]. In this case, the expression of a semi-dominant *gai* mutant (a *DELLA* gene which negatively regulates the gibberellic acid signaling pathway) under an anther- and pollen-specific promoter resulted in male sterility that was reverted by kinetin.

Type-B ARR binding sites are found associated with genes involved in biosynthesis, inactivation, and signaling (Figure 2). Based on gene expression data, type-B ARR targets upregulated by cytokinin include three genes involved in gibberellic acid biosynthesis (*GA3OX1*, *GA2*, and *GIM2*), whereas expression of the *GID1b* receptor is repressed. Thus, cytokinin-gibberellic acid crosstalk points to a complex set of interactions between these two phytohormones; the type-B ARRs interact with DELLAs to facilitate the cytokinin transcriptional response and oppose gibberellin activity [99], but the targets of the type-B ARRs include genes that will induce gibberellic acid biosynthesis on the one hand but reduce gibberellin perception on the other hand.

### 3.6. Cytokinin-Ethylene Crosstalk

Ethylene is a gaseous hormone that regulates cell proliferation and expansion, senescence, fruit ripening, and responses to biotic and abiotic stresses [101,102]. Cytokinin and ethylene often function antagonistically in the shoot, with cytokinin being associated with greening and cell proliferation and ethylene with aging processes such as ripening and senescence and the inhibition of cell proliferation [2,3,101,103,104]. On the other hand, the two hormones operate cooperatively in processes such as the regulation of root growth, where both hormones serve to inhibit root growth through effects on cell proliferation and cell elongation [105,106,107,108,109,110]. Significantly, cytokinin positively stimulates ethylene biosynthesis by activating ACC synthases (ACSs) via both transcriptional and post-transcriptional mechanisms [89,106], the production of ethylene facilitating the ability of cytokinin to inhibit hypocotyl elongation in dark-grown seedling as well as to inhibit root growth [89,106,107,108,109].

Type-B ARR binding sites are found to be associated with genes involved in ethylene biosynthesis and signal transduction (Figure 2); note that ethylene does not require specific mechanisms for inactivation or transport due to the fact of its gaseous nature and ability to diffuse across cell membranes. Two *ACS* genes are identified, *ACS2* expression being upregulated by cytokinin, consistent with cytokinin-activating ethylene biosynthesis in a type-B ARR-dependent manner [105,106]. Additionally, the type-B ARRs bind to many genes that affect ethylene signal transduction. Most of these encode elements that modulate activity of the signaling pathway, rather than being within the pathway itself, although the ethylene receptor ETR2 was identified as a target that has an expression which is downregulated by cytokinin. The modulating factors include *ARGOS* and *ARL* from the *ARGOS* gene family which are transmembrane proteins that act at the level of the receptors and desensitize the plant to ethylene [104,111,112]; *TRP1* which functions as a positive regulator of ethylene signaling potentially by binding to receptors and interfering with their ability to interact with CTR1 [113]; and *ETP* and *EBF* genes which encode F-box proteins involved in the degradation of EIN2 and EIN3, respectively [114,115,116].

### 3.7. Cytokinin-Jasmonic Acid Crosstalk

Jasmonic acid is an important regulator of plant development and stress responses, influencing flower development and senescence, as well as responses to herbivorous insects, necrotrophic pathogens, and wounding [117,118,119]. Generally, cytokinin and jasmonic acid are viewed as antagonists; as such, they have opposing effects on callus growth, chloroplast functionality, and xylem development [37,120,121,122]. However, cytokinin accelerates the production and release of jasmonic acid in wounded plants, and the application of jasmonic acid to potato stem node cultures increases the amount of biologically active cytokinin, pointing to the complex nature of cytokinin–jasmonic acid interactions [123,124,125].

Type-B ARR binding sites are found to be associated with genes involved in biosynthesis, degradation/inactivation, and signaling (Figure 2). Expression for many of these genes is upregulated by cytokinin. This includes multiple genes involved in jasmonic acid biosynthesis, consistent with cytokinin being able to induce biosynthesis of jasmonic acid in the wound response [123]. On the other hand, the most pronounced effect with jasmonic acid signaling is the upregulation of many members of the JAZ family, negative regulators of the jasmonic acid response, suggesting that cytokinin may reduce sensitivity to the jasmonic acid response. Of potential significance, there is a higher percentage of “conserved” B-ARR candidates among the genes involved in jasmonic acid signaling (12 of 16 genes) compared to those involved in jasmonic acid metabolism (6 of 18 genes).

### 3.8. Cytokinin-Salicylic Acid Crosstalk

Salicylic acid is a phytohormone that plays a central role in plant defense against biotrophic pathogens, contrasting with jasmonic acid and its central role in resistance to necrotrophic pathogens [126,127,128]. Cytokinins also play a role in the immune response through their interactions with salicylic acid signaling. Cytokinins enhance the salicylic acid response which results in increased transcription of defense-related genes such as *SID2* for salicylic acid biosynthesis and *PR1*, a marker gene for salicylic acid responses [6,7,129,130]. This occurs because the type-B response regulator ARR2 is able to physically interact with the transcription factor TGA3 that plays a role in salicylic acid signaling. The activation of ARR2 by cytokinin and TGA3 by salicylic acid results in enhanced binding of this transcription factor complex to defense genes such as *PR1* [6,129].

Analysis of type-B ARR ChIP-seq data suggests that additional mechanisms may also play a role in mediating crosstalk between cytokinin and salicylic acid, because type-B ARR binding sites are found associated with genes involved in the metabolism, transport, and signaling of salicylic acid (Figure 2). Perhaps the most interesting of these is *EDS5*, a “common” target that is induced by cytokinin. EDS5 is a MATE-transporter involved in the movement of salicylic acid from the chloroplasts, where salicylic acid biosynthesis occurs, to the cytosol and its induction by cytokinin could thus facilitate salicylic acid activity [127,131]. Many genes encoding key signaling elements are also among the B-ARR candidate targets, although there is minimal information as yet about the role of cytokinin in their regulation. These include NPR1, a transcriptional co-activator, the NPR1-interacting TGA transcription factors, the histone acetyltransferase *HAC5*, and the NPR1-suppressor NIMIN1 [126,127,128]. These findings open new possibilities for cytokinin to participate in plant responses to pathogens through the regulation of salicylic acid activity.

## 4. Conclusions

As described above, analysis of the type-B ARR ChIP-seq datasets reveals that one mechanism for cytokinin crosstalk with other phytohormones is the transcriptional regulation of elements involved in their metabolism, transport, and signal transduction [24,34]. That these represent primary targets of the type-B ARRs emphasizes the significance of such crosstalk in modulating hormone responses in plants. By integrating transcriptome studies with the ChIP-seq studies, type-B ARR target genes are identified which have expressions that rapidly change in response to cytokinin, pointing to where the cytokinin response is initially “felt” by other phytohormones. Many genes are associated with highly reproducible type-B ARR binding sites but exhibit no apparent change in expression in response to cytokinin treatment. Many of these are likely to also be of physiological significance but do not appear in the cytokinin transcriptome datasets for a variety of reasons. First, some may represent genes that exhibit slower kinetics or require higher hormone concentrations for their transcriptional response to cytokinin and, thus, are missed in the short-term transcriptome studies. Consistent with this possibility are the pronounced changes in cytokinin-dependent gene expression found in long-term transcriptomic studies as well as the increases in ARR1 binding sites found by ChIP-seq when comparing a 24 h to a 4 h cytokinin treatment [34,42]. Second, some candidate genes may exhibit a cytokinin response only under specific conditions or in certain tissues. Consistent with this possibility is the identification of the key shoot meristem regulator *WUSCHEL* as a type-B ARR target transcriptionally induced by cytokinin, although it does not generally appear in transcriptomic studies [24,34,43,44]. Third, expression for some candidate genes may require combinatorial interactions between the type-B ARRs and other transcription factors. Consistent with this possibility is the finding that ARR1 interacts with DELLA proteins involved in gibberellic acid signaling and that ARR2 interacts with TGA3 involved in salicylic acid signaling to regulate gene expression [6,99,129]. Such physical interactions between elements of phytohormone signaling pathways represent an additional mechanism by which to mediate their crosstalk. It is our hope that the type-B ARR candidate and target genes described here facilitate studies to unravel mechanisms of crosstalk between cytokinin and the other phytohormones.

## Supplementary Materials

The following are available online at https://www.mdpi.com/2223-7747/9/2/166/s1, Table S1: Conserved type-B ARR candidate genes. Table S2: Golden type-B ARR targets. Table S3: Hormone crosstalk candidates.
